# Backbone and side chain NMR assignment of the heme-nitric oxide/oxygen binding (H-NOX) domain from *Nostoc punctiforme*

**DOI:** 10.1007/s12104-022-10107-1

**Published:** 2022-09-06

**Authors:** Styliani A. Chasapi, Aikaterini I. Argyriou, Georgios A. Spyroulias

**Affiliations:** Department of Pharmacy, University of Patras, Patras, Greece

**Keywords:** H-NOX, Soluble guanylyl cyclase, sGC, NMR spectroscopy, *Nostoc punctiforme*

## Abstract

Soluble guanylate cyclase (sGC) is considered as the primary NO receptor across several known eukaryotes. The main interest regarding the biological role and its function, focuses on the H-NOX domain of the *β1* subunit. This domain in its active form bears a ferrous b type heme as prosthetic group, which facilitates the binding of NO and other diatomic gases. The key point that still needs to be answered is how the protein selectively binds the NO and how the redox state of heme and coordination determines H-NOX active state upon binding of diatomic gases. H-NOX domain is present in the genomes of both prokaryotes and eukaryotes, either as a stand-alone protein domain or as a partner of a larger polypeptide. The biological functions of these signaling modules for a wide range of genomes, diverge considerably along with their ligand binding properties. In this direction, we examine the prokaryotic H-NOX protein domain from *Nostoc punctiforme* (*Npun* H-NOX). Herein, we first report the almost complete NMR backbone and side-chain resonance assignment (^1^H, ^13^C, ^15^ N) of *Npun* H-NOX domain together with the NMR chemical shift-based prediction of the domain’s secondary structure elements.

## Biological context

Soluble guanylate cyclase (sGC) is a widely known signaling molecule transducing signals mediated by the first messenger, a nitric oxide (NO). This NO sensor is largely found across the eukaryotic species, and it is responsible for vasodilation and neurotransmission in mammals (Papapetropoulos et al., 2015). Enzyme’s biological role and function focus on the H-NOX domain of the *β1* subunit. The active form of H-NOX domain bears a heme molecule as natural ligand, which binds the NO and other diatomic gases. Upon binding of NO to the H-NOX domain of the sGC *β1* subunit, sGC catalyses the conversion of guanosine 5-triphosphate (GTP) to the second sequential signal molecule, cyclic 3,5-guanosine monophosphate (cGMP) (Stone and Marletta, 1996). Disorders in its normal functioning or even the interruption of the signaling pathway result in a variety of pathological conditions (hypertension, strokes, erectile dysfunction, chronic renal failure, etc.). Multiple isoforms of sGC exist in humans, including sGC forms with *α1*, *α2*, *β1* and *β2* (Mayer and Koesling, 2001). sGC subunits *α1/α2* share 46% sequence homology, while *β1/β2* subunits share 41% sequence homology. Isoform *α1β1* is the most studied, while the role of other isoforms is poorly understood, although the *α2β1* complex is located in neural cells. NO receptors are found not only in mammalian cells but also in processes of bacteria across many phyla such as *Cyanobacteria*, *Proteobacteria*, *Thermotogae*, *Firmicutes* and *Bacteroidetes* (Boon and Marletta, 2005; Guo et al., 2018; Karow et al., 2004). The correlation between the NO signaling in humans and bacterial NO sensing was first discussed by the (Nioche et al., 2004), and (Pellicena et al., 2004) research groups, providing information about their homology ~ 15–40% in comparison to the human H-NOX domain. The detection-binding of NO by the homologous bacterial H-NOX domains plays a key role in the regulation of bacterial metabolism and in the formation of biomembrane (Plate and Marletta, 2012).

**Fig. 1:**
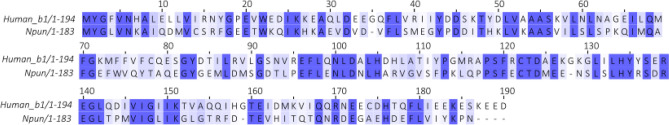
Sequence alignment of the Human sGC *β1* H-NOX domain and Nostoc punctiforme H-NOX domain. Amino acid numbering is according to the *β1* sequence of human H-NOX protein domain. Color coding was selected to highlight the conserved residues with dark blue, the conserved type of residues with consensus identity > 30% with light blue and the non-conserved residues with white color

The available structural studies by X-ray crystallography do not indicate significant changes between the various complexes of bacterial H-NOX domains, either in complexes with or without diatomic gases (practically all available models show significant similarity with RMSD < 0.3Å (Makrynitsa et al., 2019). Thus, NMR conformational and dynamic studies of other bacterial H-NOX domains are gaining interest indicating sites of interaction and other conformational modifications shedding light to analogous mechanisms of action in the human H-NOX domain (Makrynitsa et al.^2022^, ^2021^; Argyriou et al., 2021; Erbil et al., 2009). Lately, structural studies have been reported regarding the NMR conformational data of *Nostoc sp*. H-NOX domain, as well as interaction studies of this bacterial H-NOX domain with NO and known stimulators of human sGC H-NOX domain. *Npun* shares 56.10% sequence homology with *Nostoc sp.* H-NOX domain and each one shares 39.89% (Fig. 1) and 33.86% with the human H-NOX domain respectively. Hence, *Npun* H-NOX is offered as a fine descriptive protein model for NMR conformational examination, analysis, and comparison with the so far reported studies.

The present study reports the first backbone and side-chain assignment of the *Npun* H-NOX domain (1–183 residues) and the secondary elements’ prediction based on the assigned NMR chemical shifts in solution. This work will help to understand better the recognition mechanism of bacterial H-NOX domains’ discrimination and selectivity towards diatomic gases. This in turn, will unravel the role of the different and conservative amino acids in the identification process and binding of diatomic gases. Exploration of the structural and conformational changes of the H-NOX domains and the way which they differentiate among the various organisms (facultative versus obligate aerobes) may shed light on their biological role.

## Methods and experiments

### Protein sample preparation

**Fig. 2:**
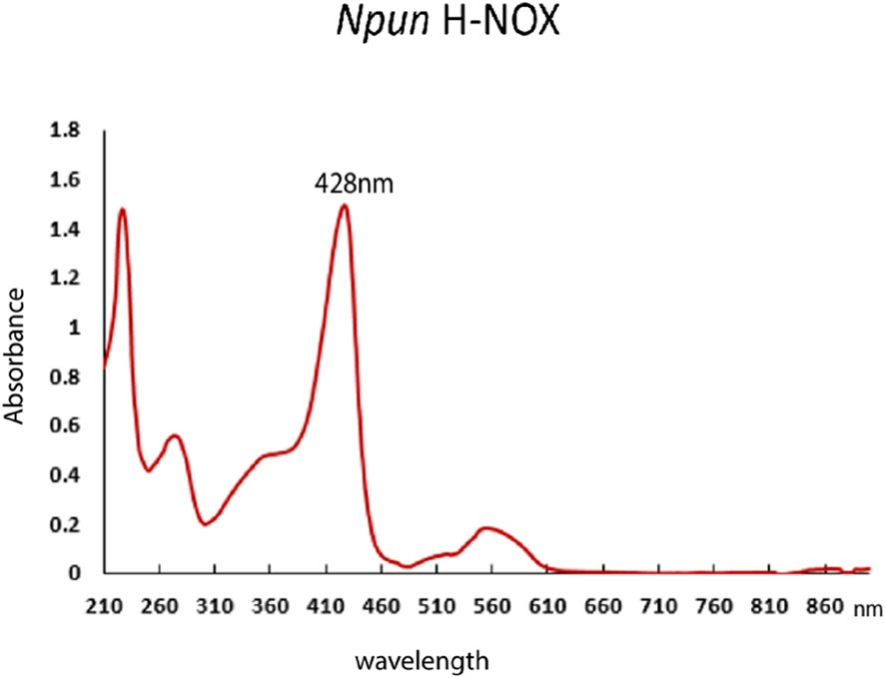
UV–visible absorption spectrum indicating the Fe(II) heme of *Npun* H-NOX domain at 428 nm

H-NOX domain from *Nostoc punctiforme* was cloned and expressed using the pET-22b ( +) expression vector. The H-NOX protein domain comprising the residues 1–183 of the *Npun* H-NOX domain expressed in *Escherichia coli* BL21 (DE3) Star cells. The bacteria were grown in minimal medium (M9) containing ^15^NH_4_Cl (1 g/L) and ^13^C-glucose (4 g/L) for isotopic labelling, 0.5 mM aminolevulinic acid (δ-ALA) for enhancement of heme molecule production and ampicillin (1 mg/L) for bacterial selection. The culture was incubated in 37 °C (220 rpm) until the OD_600_ was approximately 0.8, after 0.5 mM IPTG induction culture incubated overnight at 18 °C (180 rpm). The protein purification procedure performed using an ion exchange column (GE Healthcare) using a NaCl gradient buffer solution. Finally, size exclusion chromatography performed using a Superdex 200 10/300GL column on an AKTA purifier 10 FPLC system (GE Healthcare). Prior to NMR analysis a 10% of 99.9% D_2_O was added to protein samples resulting in a concentration of 0.6 mM. UV–Vis absorption spectrum has a Soret band at 428 nm which is indicative of a Fe(II) five-coordinate heme complex (Fig. 2). These data, along with the other reports on bacterial H-NOX, strongly suggest that the *Npun* H-NOX protein domain is in the Fe(II) – H-NOX diamagnetic complex form (Boon et al., 2006; Tsai et al., 2012; Dai et al., 2012; Alexandropoulos et al., 2016; Makrynitsa et al., 2022).

### NMR spectroscopy

The selected solvent system for all NMR experiments is the 90% H_2_O − 10% D_2_O. NMR experiments acquired at 298 K on a Bruker Avance III High Definition four-channel 700 MHz NMR spectrometer, equipped with a cryogenically cooled 5 mm ^1^H/^13^C/^15^ N/D Z-gradient probe (TCI). The NMR experiments for the assignment of the selected protein-domain sequence were collected as follows and followed the standard methodology. Backbone assignments for Npun H-NOX domain was obtained from the analysis of the following heteronuclear two-dimensional (2D) and three-dimensional (3D): 2D [^1^H-^15^ N] HSQC and 2D [^1^H-^13^C] HSQC, 3D HNCO, 3D HN(CA)CO, 3D TROSY-HN(CO)CACB, 3D TROSY-HNCACB, 3D HBHA(CO)NH, aliphatic 3D (H)CCH-TOCSY, 3D HNHA, 3D HNCA, 3D ^15^ N-NOESY, 3D ^13^C-NOESY aliphatic and aromatic (Davis et al., 1992, Zhang et al., 1997 and Bax and Grzesiek 1993). Additionally, a set of 3D CBCA(CO)NH modified NMR experiments were recorded to select the sequential neighbours of residues without aliphatic ^13^Cγ atom such as Ala, Gly, Ser, Asp, Asn, Cys and the aromatic residues, or amino acids lacking a γCO group (Ala, Ser, Cys and aromatic residues). The acquired NMR data were processed using the TopSpin 3.5 pl7 software and analysed with CARA 1.9.1.7. (Keller 2004).

## Extent of assignment and data deposition

**Fig. 3:**
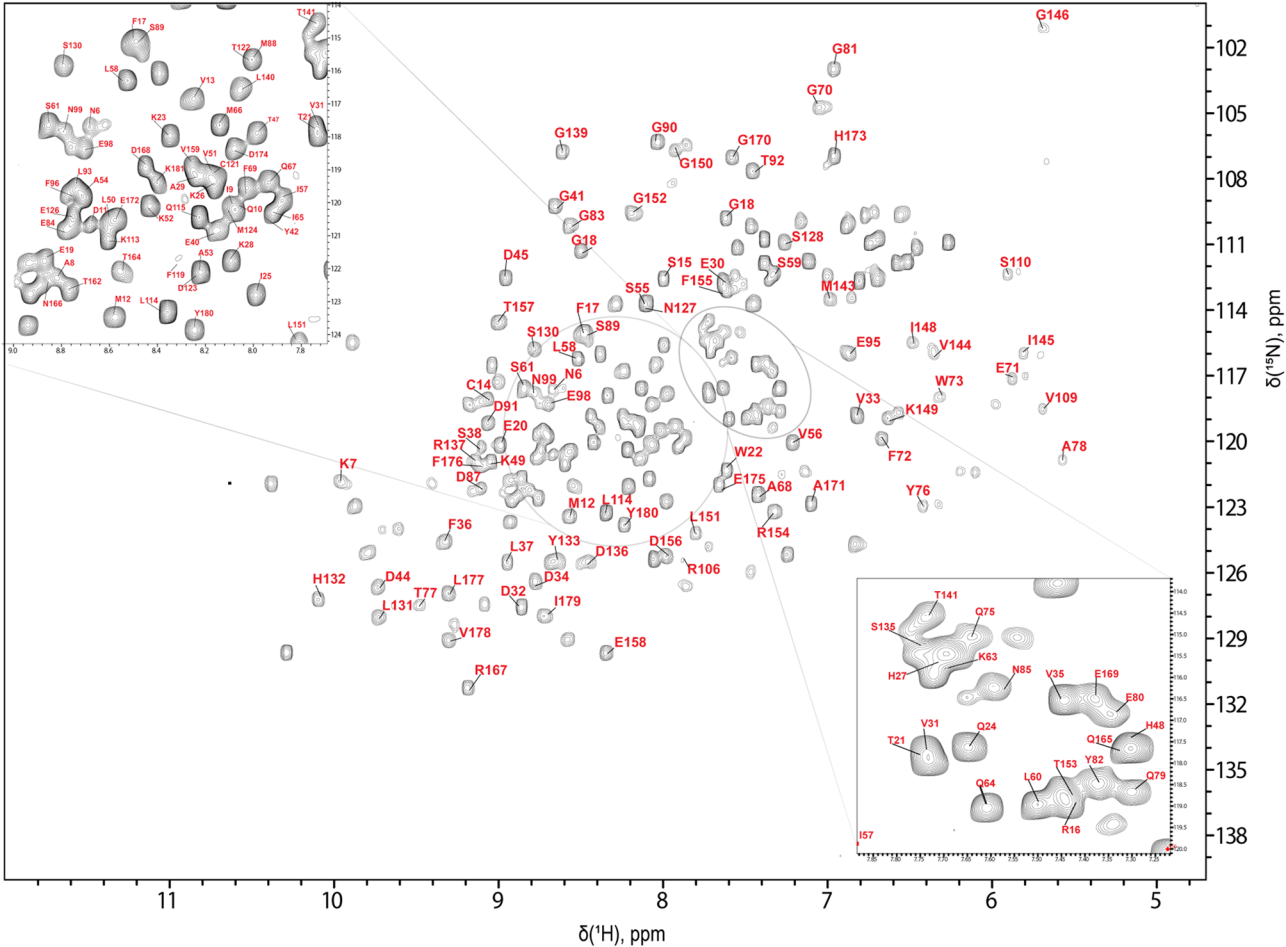
700 MHz 2D ^1^H-^15^ N HSQC NMR spectrum of *Npun* H-NOX at 298 K. Resonance assignments are labelled in red according to the native sequence of *Nostoc punctiforme* H-NOX domain. The circled areas are magnified to the adjacent squared windows to reveal the crowded central regions

The 2D ^1^H-^15^ N HSQC NMR spectrum of *Npun* H-NOX shows amide signals with good dispersion, indicating a properly folded tertiary structure of the protein domain in solution. In Fig. 3 are depicted all the dispersed NH signals in the 2D ^1^H-^15^ N HSQC NMR spectrum. Analysis of the NMR spectral set resulted in the 85% assignment of ^1^H/^15^ N backbone pairs and 89.4, 87.7, 65.4, 89.5 and 89% of all Hα, Hβ, CO, Cα and Cβ chemical shifts of the *Npun* H-NOX. However, no backbone amide signals were detected for M1, Y2, G3, L4, L100, D101, N102, L103, H104, V107, F111, S118, L129, H160, N183. The eight Proline residues also are absent from the 2D ^1^H-^15^ N HSQC since they don’t bear the characteristic amide proton. However, assignment of the side chain for five (P43, P62, P94, P112 and P142) from the eight prolines was conducted only through the direct ^13^C detection experiments from the 2D ^1^H-^13^C HSQC and ^1^H-^1^H TOCSY NMR spectra. The chemical shift values for each of the assigned atoms have been deposited in the Biological Magnetic Resonance Bank (https://bmrb.io/) under the accession no. 51495. These assignment percentages are reasonably comparable to those of *Ns* H-NOX in its native (heme-bound) state of (Alexandropoulos et al., 2016), further corroborating their high sequential homology. Additionally, based on Cα, CO and side chain resonances the 15 amino acids V5, I46, V74, L86, L97, A105, G108, E120, E125, Y133, R134, E138, L147, I161 and Q163 were detected and assigned. In total, 30 amino acids out of 183 could not be assigned unambiguously and the vast majority are located in or form the heme pocket. As a consequence, the high flexibility of the heme and the Fe(II)-H-NOX coordination state (5-coordination complex), which is also noted to other related bacterial H-NOX domains, may have an effect on peak broadening (beyond detection) due to conformational exchange among the multiple poses adopted by the 5-coordinated heme into its cavity. All the above corroborate with the phenomenon of the large number of missing peaks (Erbil et al., 2009; Alexandropoulos et al., 2016; Makrynitsa et al., 2021; Chen et al., 2021).

Amino acids L100-H104 and G108 could not be identified and the V74, L86, L97, A105 and V107 residues lack of H^N^ and N assignments. These amino acids are considered part of the two *α*-helices which are placed above and beneath the heme prosthetic group. These H-NOX sequence fragments have been already discussed and experimentally confirmed from the NMR and the crystal structures of *Shewanella oneidensis* (PDB: 2KIL, 2KII, 4U99) and *Caldanaerobacter subterraneus* H-NOX domains (PDB: 1U55) (Herzik et al., 2014; Erbil et al., 2009; Pellicena et al., 2004). In detail, the residues L86 and L97 in *So* H-NOX structure appears to be placed in positions where they are the last residues forming an *α*-helix and turning to a random coil structure. Additionally, the amino acid V74 seems to be part of the *α*-helix beneath the heme but also the one residue approaching closest to the heme. These three amino acids, V74, L86 and L97, seem to contribute to the same secondary elements in *Npun* H-NOX as well.

Identification of the proline residues cis/trans conformation was based on the analysis of the 3D TROSY HNCACB and the aliphatic 3D (H)CCH-TOCSY. Examination of the chemical shift difference of the ^13^Cβ and ^13^Cγ atoms of the identified proline residues was conducted based on the Δβγ = δ[^13^Cβ]- δ[^13^Cγ] equation (Schubert et al., 2002). The comparison resulted to a trans conformation of the five identified prolines of the *Npun* H-NOX domain.

**Fig. 4:**
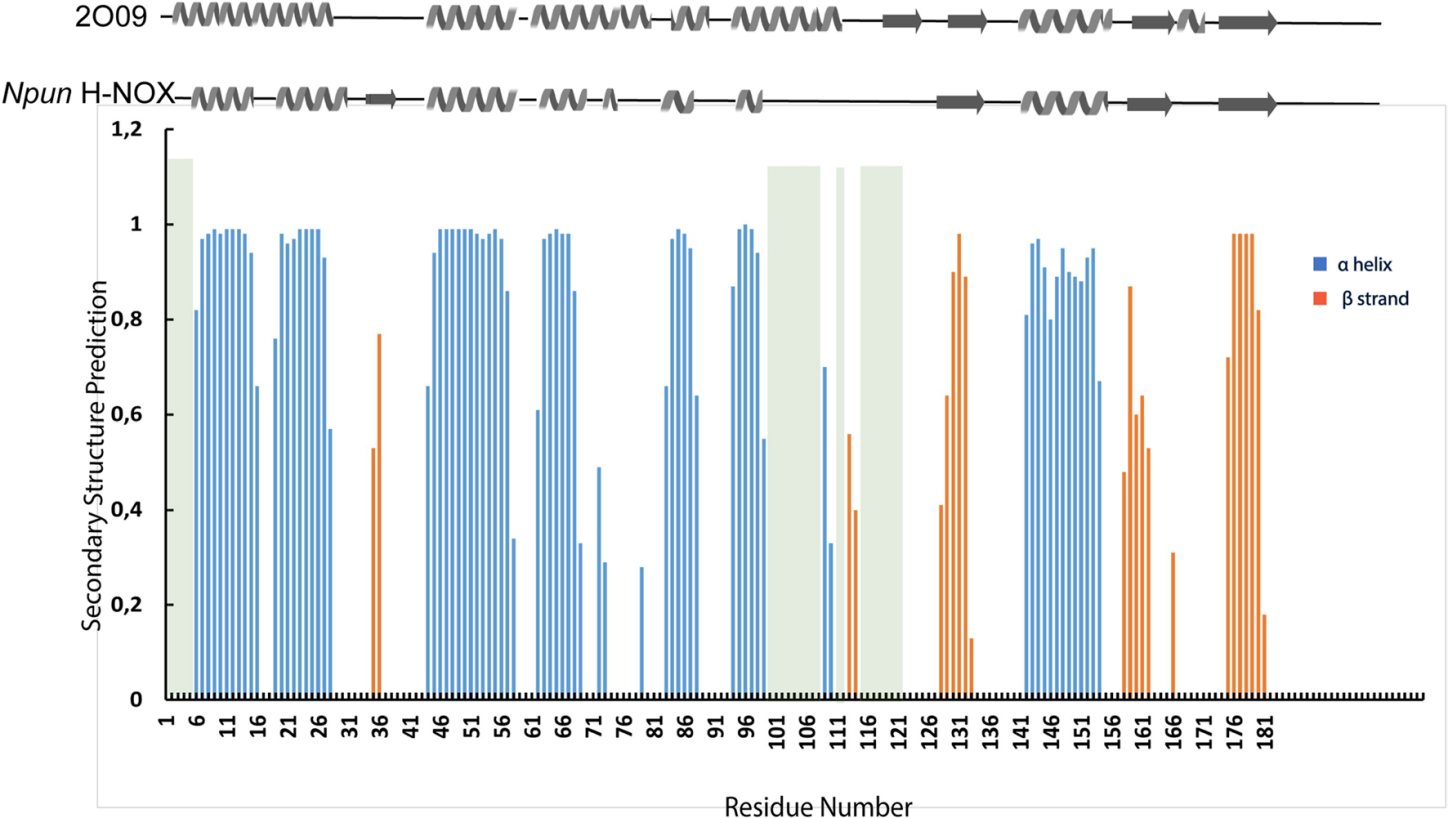
Secondary structure of the heme bound *Npun* H-NOX as predicted by TALOS + from the chemical shift values. Color coding indicate orange for *β*-sheets and blue for *α*-helix, while grey bars point out the unassigned sequence part. Cartoon illustrations of the secondary structure elements from the Ns H-NOX X-ray structure (PDB: 2O09) and the Npun H-NOX as resulted from TALOS + prediction, are placed on top. The secondary structure elements of the Ns H-NOX X-ray structure obtained using the pdbsum (Laskowski et al., 1997)

Secondary structure prediction of the *Npun* H-NOX domain was performed using the chemical shift assignments of the atoms H^N^, N, Hα, CO, Cα and Cβ, for each amino acid in the sequence using TALOS + server (Shen et al., 2009). TALOS + prediction results indicate that secondary structure elements are composed from 7 *α*-helices and 4 *β*-strands organized in a *αaβααααβαββ* topology (Fig. 4). However, in TALOS + prediction there are two residues, 35 and 36, indicating the existence of an extended strand (E). The unassigned sequence part which comprises the residues L100-G108 is forming more likely an *α*-helix as it is indicated from the TALOS + prediction results for the two residues beta strand around the residues 112, as it is also reported in the X-ray structure where in *Npun* H-NOX domain is the area with the unassigned residues similar to *Nostoc sp.* H-NOX domain (Alexandropoulos et al., 2016),(Makrynitsa et al., 2021). Whereas the unassigned, annotated in grey bars in Fig. 4, residues116-118 seem to form a loop or the 118 to be the initial residue of a *β*-strand, as depicted in the related NMR structures of *So* H-NOX (PDB: 2KII, 2KIL), *Sw* H-NOX (PDB: 6OCV) and human b1 H-NOX (PDB: 5MNW). Summarizing, the present work describes the NMR study in solution of the *Nostoc punctiforme* H-NOX domain, which shares 38% sequence identity with the human sGC H-NOX domain. This analysis is a result of 3D triple resonance NMR experiments on the *Npun* H-NOX protein samples after following established molecular biology protocols. Signals’ dispersion of the ^1^H-^15^ N HSQC spectrum indicates a well-folded protein domain, with the almost complete sequence-specific assignment of the protein resonances revealing a mixed *α/β* secondary structure elements similar to *Nostoc sp.* H-NOX domain. The high similarity of the structural elements forming the heme cavity across many studied bacterial H-NOX domains is of great biological significance since they might act synergistically defining the ligands selectivity according to their organism functionality and biological role. Hence, the present system is of additive value to the so far similar NMR structural studies and can be exploited for comparative analysis regarding the redox switching mechanism of heme, the coordination properties of the heme iron along with the dynamics of the H-NOX domain under ligand-binding or gas sensing conditions.

## Data Availability

Assignment deposited in BMRB with ID: 51495.
